# N-Glycosylation Alteration of Serum and Salivary Immunoglobulin A Is a Possible Biomarker in Oral Mucositis

**DOI:** 10.3390/jcm9061747

**Published:** 2020-06-05

**Authors:** Enikő Gebri, Zsuzsanna Kovács, Brigitta Mészáros, Ferenc Tóth, Ádám Simon, Hajnalka Jankovics, Ferenc Vonderviszt, Attila Kiss, András Guttman, Tibor Hortobágyi

**Affiliations:** 1 Department of Dentoalveolar Surgery and Dental Outpatient Care, Faculty of Dentistry, University of Debrecen, Nagyerdei krt. 98., H-4032 Debrecen, Hungary; gebri.eniko@dental.unideb.hu; 2 Horváth Csaba Laboratory of Bioseparation Sciences, Research Center for Molecular Medicine, Doctoral School of Molecular Medicine, Faculty of Medicine, University of Debrecen, Nagyerdei krt. 98., H-4032 Debrecen, Hungary; zsuzsa19890608@gmail.com (Z.K.); brigi.meszi@gmail.com (B.M.); simonadam9085@gmail.com (Á.S.); andrasguttman@gmail.com (A.G.); 3 Research Institute of Biomolecular and Chemical Engineering, University of Pannonia, Egyetem u 10., H-8200 Veszprém, Hungary; jankovicsh@almos.uni-pannon.hu (H.J.); von007@almos.uni-pannon.hu (F.V.); 4 Department of Biomaterials and Prosthetic Dentistry, Faculty of Dentistry, University of Debrecen, Nagyerdei krt. 98., H-4032 Debrecen, Hungary; ferenc.toth@dental.unideb.hu; 5 Department of Hematopoietic Transplantation Centre, Faculty of Medicine, University of Debrecen, Nagyerdei krt. 98., H-4032 Debrecen, Hungary; akiss@med.unideb.hu; 6 Institute of Pathology, Faculty of Medicine, University of Szeged, Állomás utca 1., H-6725 Szeged, Hungary; 7 MTA-DE Cerebrovascular and Neurodegenerative Research Group, Department of Neurology, Faculty of Medicine, University of Debrecen, Nagyerdei krt. 98., H-4032 Debrecen, Hungary; 8 Institute of Psychiatry Psychology and Neuroscience, King’s College London, De Crespigny Park, London SE5 8AF, UK

**Keywords:** autologous peripheral stem-cell transplantation (APSCT), glycomics, glycoprotein, immunoglobulins, IgA, oral immunity, oral mucositis

## Abstract

Background: Oral and enteral mucositis due to high-dose cytostatic treatment administered during autologous and allogeneic stem-cell transplantation increases mortality. Salivary secretory immunoglobulin A (sIgA) is a basic pillar of local immunity in the first line of defense. Altered salivary sialoglycoprotein carbohydrates are important in the pathologies in the oral cavity including inflammation, infection and neoplasia. Therefore, we assessed whether changes in the salivary and serum IgA glycosylation correlated with development and severity of oral mucositis. Methods: Using capillary electrophoresis, comparative analysis of serum and salivary IgA total N-glycans was conducted in 8 patients with autologous peripheral stem-cell transplantation (APSCT) at four different stages of transplantation (day −3/−7, 0, +7, +14) and in 10 healthy controls. Results: Fourteen out of the 31 structures identified in serum and 6 out of 38 in saliva showed significant changes upon transplantation compared with the control group. Only serum core fucosylated, sialylated bisecting biantennary glycan (FA2BG2S2) showed significant differences between any two stages of transplantation (day −3/−7 and day +14; *p* = 0.0279). Conclusion: Our results suggest that changes in the serum IgA total N-glycan profile could serve as a disease-specific biomarker in patients undergoing APSCT, while analysis of salivary IgA N-glycan reflects the effect of APSCT on local immunity.

## 1. Introduction

Comprehensive analysis of the carbohydrate moiety of glycoproteins offers new paths for biomarker research [1,2]. Glycosylation is essential for the functions of immunoglobulins, such as secretory immunoglobulin IgA (sIgA) dimerization, polymeric Ig receptor-mediated transcytosis, and adhesion of pathogens to the mucosal surface, and is responsible for antibody binding to the mucus layer [3]. Several saliva components protect the mucosa. Salivary sIgA is crucial in immune exclusion via direct interaction with microbial antigens, and eliminates viruses by non-virulent immune complex formation, whereby N-glycan sialic acids of sIgA inhibit sialic acid-binding viruses. It also neutralizes bacterial lipopolysaccharide (LPS), and maintains commensal homeostasis, thereby preventing disseminating pathogens [4]. IgA in the serum also has an anti-inflammatory role [5], especially in cases when systemic immunity weakens. In particular, as a result of chemotherapy, there is decreased sIgA secretion in acute leukemia. In oral mucositis (OM), which is a common complication of the cytostatic treatment, there is a further decrease in IgA secretion [6]. The attenuated antimicrobial activity is partly due to the reduced antioxidant capacity, which increases mucosal sensitivity to infections and tissue injury [7]. High-dose cytostatic therapy administered as part of autologous peripheral stem-cell transplantation (APSCT) in hematological malignancies often causes severe oral and enteral mucosal barrier injury. As a consequence, dissemination of pathogens and fatal sepsis may ensue. In less severe cases, mucositis increases the length of hospitalization, enhances susceptibility to further infections and significantly worsens quality of life. During APSCT, serum IgA, which has a diverse role in mucosal immunity, also decreases [8]. While serum immunoglobulin A (IgA) usually returns to the normal level within six or seven months, salivary sIgA level needs up to five years to recover, probably due to the persistence of weak mucosal immunity [7]. The biochemical and immunochemical properties of serum and secretory IgA are different. Therefore, the sIgA glycosylation pattern could be suitable as a possible biomarker to monitor pathological processes in the oral cavity [3].

**Figure 1:**
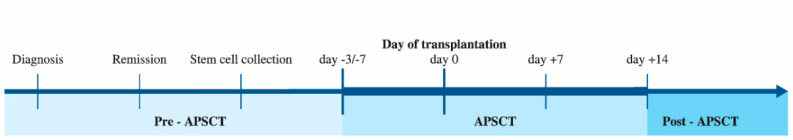
Study design. Timeline of autologous peripheral stem cell transplantation (APSCT). Saliva and serum sampling were performed at the four defined time-points of APSCT (day −3/−7, day 0, day +7, day +14). Timescale pre-and post APSCT were different among the individual cases (i.e., the thin line is not proportional to the elapsed time). Day −3/−7 is the time of admission and start of the conditional therapy; day 0 is the day of the transplantation (administering the harvested CD34+ stem cells in a stem cell infusion); day +7 is usually the deepest point of cytopenia with most severe oral mucositis; by day +14, neutrophil and thrombocyte engraftment has developed if insertion and proliferation of stem cells were successful.

Our aim was to examine the effect of high-dose intensive immunosuppressive therapy on local immunity in the context of APSCT (Figure 1). We tested whether the N-glycosylation patterns of both serum and salivary IgA at different stages of APSCT could serve as possible biomarkers of OM, even in subclinical cases. This would enable early diagnosis, more effective therapy and disease monitoring.

## 2. Materials and Methods

### 2.1. Study Groups and Ethics

Collection of serum and saliva samples was carried out at the institutional hematopoietic transplantation centre from 8 patients admitted for APSCT due to malignant hematological disease, and at the dental outpatient clinic from 10 healthy controls between 7 and 8 a.m. The use of human subjects followed an approved protocol and satisfied the requirement of the IRB (ethical approval UD 5570-1/2018/EKU). All subjects gave their informed consent for inclusion before they participated in the study. The study was conducted in accordance with the Declaration of Helsinki.

### 2.2. Patient Characteristics and Collection of Serum Samples

Eight patients (5 females and 3 males) with malignant hematological disease who required APSCT were included in the study. Average age was 49.5 ± 14.19 years. Reason for APSCT was non-Hodgkin lymphoma (NHL) in 3 patients, Hodgkin lymphoma (HL) in 2, and multiple myeloma (MM) in 3 cases. Five patients were in complete morphologic remission (CMR), one in very good partial remission (VGPR) and 2 in partial remission (PR) prior to transplantation. Serum and saliva samplings were performed at the same time on specific days of the peritransplantation period as follows: day of hospital admission (day −3/−7), day of transplantation (day 0) and day +7 and day +14 post-transplantation. OM grade was established according to WHO criteria [9]. As a control group, 10 age-and sex-matched patients were selected (average age: 41.9 ± 18.35 years, 9 females and 1 male). Mann–Whitney *t*-test (*p* = 0.2645) showed no statistically difference between the control and the transplanted group. For more details of patients’ demographics see Table S1. The conditioning regimen was BEAM (BCNU, etoposide, cytosine arabinoside, melphalan) protocol in Hodgkin and non-Hodgkin lymphoma prior to the transplantation [9], while in MM it was high-dose melphalan (≥200 mg/m^2^) [9]. Patients with severe chronic disease (diabetes, autoimmune diseases, acute or chronic inflammatory diseases, etc.) and previous malignancy were excluded from the study. Patients in both groups were free of dental foci (dental calculus, radices, etc.) at the time of sampling. Study design was aligned with STROBE recommendations [10] and, using sample size calculator Sampsize (epiGenesys, Sheffield, UK), it was a pilot study [11]. Power values were in the range of 59–99% with median 94% using G-power 3.1.9.2. software (Informer Technologies Inc., Düsseldorf, Germany). Bone marrow biopsy examination, qualitative and quantitative analysis of peripheral blood samples and measurement of serum immunoglobulin levels were performed at admission (day −3/−7). Results were in the normal range in each patient and immunoglobulin A levels in particular were between 0.85 g/L and 3.2 g/L (reference range: 0.7–4.00 g/L). This indicates that the plasma cell repertoire was not affected prior to transplantation. Serum samples were collected using clot activator containing serum tubes (BD Biosciences, Franklin Lakes, NJ, USA). The collected blood samples were centrifuged at 7500× *g* for 30 min and the serum fractions were stored at −70 °C one hour after collection until further processing.

### 2.3. Collection of Unstimulated Whole Saliva (UWS)

Saliva collection was performed according to the standard methods [12]. Both controls and patients were in a sitting position during the sampling with eyes open and a slightly tilted head. Following oral cavity rinse with 25 mL of physiological saline solution (B. Braun Melsungen AG, Melsungen, Germany) for 30 s, saliva was collected for 5 min in an externally pre-disinfected 15 mL lockable Falcon tube (Sigma-Aldrich, St. Louis, MO, USA). Participants adapted to the test condition for 5 min prior to sample collection. Taking into account the diurnal variation of saliva constituents, samplings were done at a specified time window: between 7 a.m. and 8 a.m., one hour after eating, drinking, or tooth-brushing in order to avoid contamination. Patients in sterile rooms used a gauze plate or DenTips (MDS096502, Medline Industries. Inc., Mundelein, IL, USA), and a disposable oral swab, impregnated with physiological saline solution, in order to maintain optimal oral hygiene during the period of cytopenia. Within one hour of collection, Halt Protease Inhibitor Cocktail (Sigma-Aldrich, St. Louis, MO, USA) was added proportionally to the saliva samples. After homogenization, saliva samples were aliquoted into 1.5 mL Eppendorf tubes and stored at −70 °C until further processing.

### 2.4. Detection of Blood Sample Immunoglobulin A (IgA) Level

Venous blood samples (5 mL) were collected into Vacutainer tubes anticoagulated with ethylenediaminetetraacetic acid (EDTA) (Vacutainer Systems, Rutherford, NJ, USA) and serum IgA levels were detected using Sysmex XN-2000 Hematology Analyzer (Sysmex Hungary, Budapest, Hungary).

### 2.5. Detection of Salivary IgA Level

After collection of saliva samples, IgA levels were measured by IDK sIgA ELISA kit (Immundiagnostik, Bensheim, Germany) according to the manufacturer’s instructions. We determined the salivary IgA secretion rate (µg/min), because it is a more stable value than IgA concentration [13].

### 2.6. Statistical Analysis

Principal component analysis (PCA) and one-way analysis of variance (ANOVA) were performed with SPSS 22 (IBM, Armonk, NY, USA) using PeakAreas% as input derived from 32 Karat software (SCIEX, Brea, CA, USA). The Shapiro–Wilk test was performed to investigate the normal distribution of data. If it passed the normality test (*p* > 0.05), ANOVA followed by Tukey post hoc test was used to compare peak intensities between experimental groups (see Supplementary Materials Tables S5 and S6), otherwise the Kruskall–Wallis test followed by Dunn’s multiple comparison was used. Differences between means at *p* < 0.05 were considered as significant. Spearman correlation analysis was performed to correlate serum or salivary IgA concentrations and flow rate with oral mucositis grades. For analysis of serum and salivary IgA ELISA results and flow rates, Mann–Whitney and Wilcoxon tests were used.

Chemicals and reagents, Z(IgA1) antibody production, expression and purification, serum and salivary IgA capturing, N-glycan release and fluorophore labeling, exoglycosidase based carbohydrate sequencing and capillary electrophoresis analysis were applied as described in detail in our recent paper [14].

## 3. Results

### 3.1. Determination of Serum and Salivary IgA Levels

**Figure 2:**
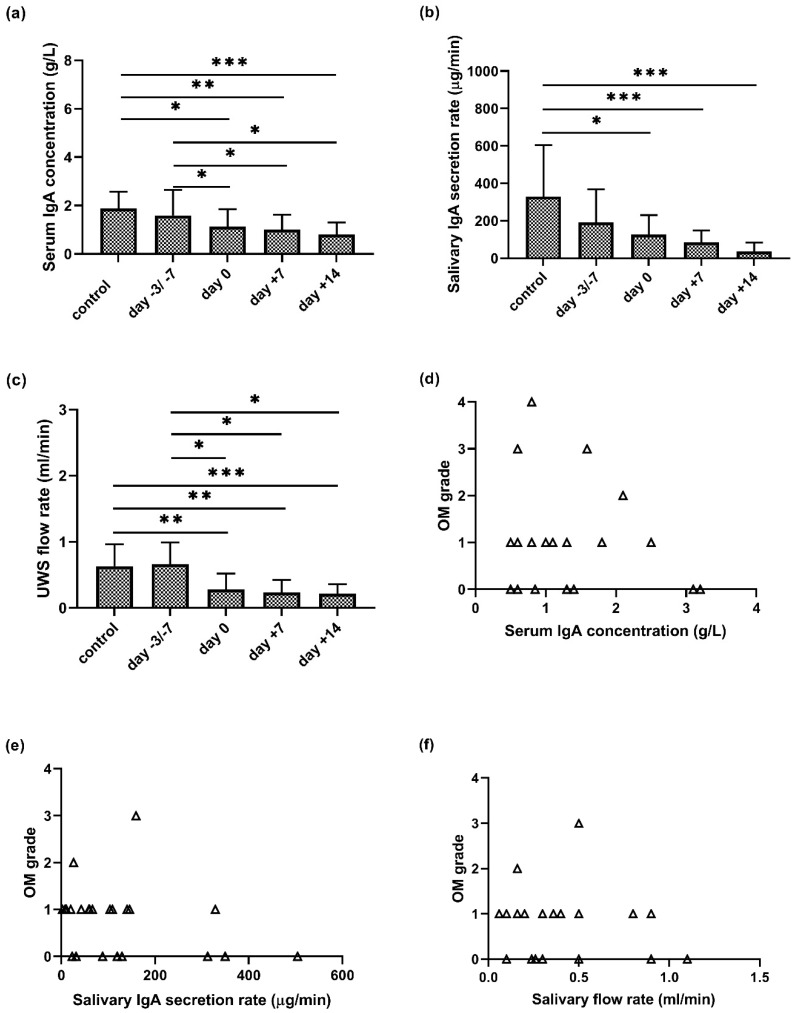
Serum IgA concentration (**a**), salivary IgA secretion rate (**b**), salivary flow rate (**c**) of unstimulated whole saliva (UWS) in the control and patient group at four stages of autologous peripheral stem cell transplantation (APSCT) and its correlation (**d**–**f**) with severity of oral mucositis (OM). In (**a–c**), the values are expressed as sample means; error bars represent the estimates of standard deviations calculated from three parallel measurements (* *p* ˂ 0.05, ** *p* ˂ 0.01, *** *p* ˂ 0.001).

There was a continuous significant decrease in serum IgA levels during APSCT (day 0, day +7, day +14) as compared to the control group (*p* = 0.024; *p* = 0.005; *p* = 0.004) and to the day of admission (*p* = 0.027; *p* = 0.028; *p* = 0.028) (Figure 2a). The IgA secretion rate was lower in the remission stage than in controls at the first sampling (day −3/−7 prior to transplantation). At the further stages of APSCT (day 0, day +7, day +14), significant differences were observed between the controls and patients (*p* = 0.015; *p* = 0.001; *p* < 0.001) (Figure 2b).

### 3.2. Unstimulated Whole Saliva (UWS) and APSCT

Contrary to expectations, the amount of UWS did not decrease in the patients in pre-APSCT remission compared to the control group. During APSCT, there was a significant decrease at day 0, day +7 and day +14 in UWS flow rate as compared to the control group (*p* = 0.008; *p* = 0.004; *p* = 0.001) and the day of admission (*p* = 0.012; *p* = 0.012; *p* = 0.012), respectively (Figure 2a).

### 3.3. Correlation of Serum and Salivary IgA Levels and Salivary Flow Rate with Oral Mucositis

The highest OM grade was variable, with grade 1 (*n* = 3), 2 (*n* = 2), 3 (*n* = 2) and 4 (*n* = 1). There was no correlation between serum IgA (g/L) or salivary IgA secretion rate (µg/min) and the degree of oral mucositis (*p* = 0.685; *p* = 0.1729) (Figure 2d,e). In contrast, there was negative correlation (*r* = −0.3622; *p* = 0.0416) between decreased salivary flow rate (mL/min) and increasing severity of OM (Figure 2f).

### 3.4. Comparison of Serum IgA N-glycome Profile of Controls and Patients Undergoing APSCT

**Figure 3:**
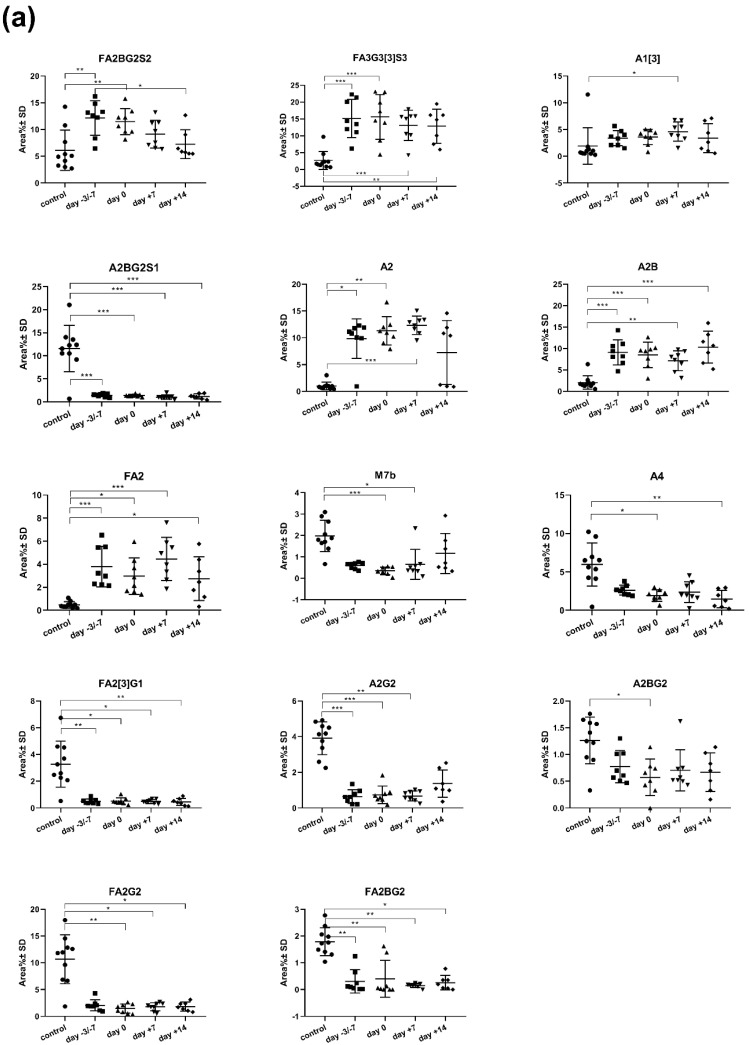

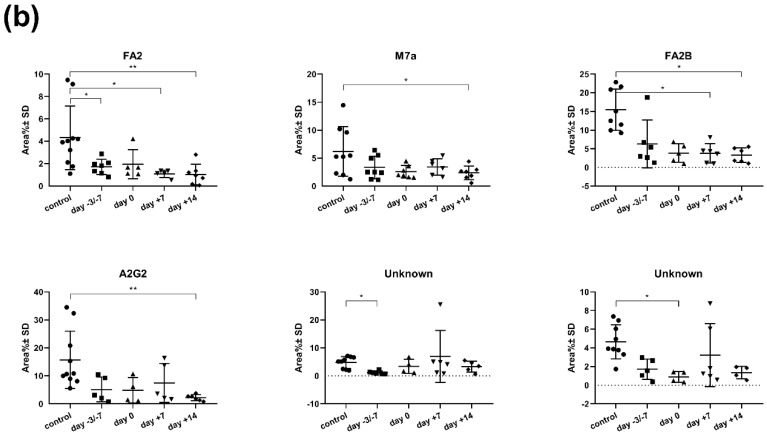
Fourteen serum (**a**) and six salivary (**b**) IgA glycoforms showed significant changes during autologous peripheral stem cell transplantation (APSCT). The abbreviations of the Oxford nomenclature were applied as referred to in our previous work [14]. The plots show the distribution pattern at various APSCT time points in patients and in controls (* *p* ˂ 0.05, ** *p* ˂ 0.01, *** *p* ˂ 0.001). For detailed results of statistical tests see also Tables S3 and S4.

Figure 3a shows the significantly changed serum IgA N-glycan structures in the control group and patient group at four stages of transplantation. The core fucosylated, sialylated bisecting biantennary glycan (FA2BG2S2) was the single significantly different structure between any two specified time points of the peritransplantation period (day −3, −7 and +14; *p* = 0.0279). Further 14 N-glycan structures showed significant differences (*p* < 0.05) between controls and any stages of APSCT. The key statistical characteristics (mean and standard errors of the PeakArea%) of the significantly changed serum IgA N-glycan structures are listed in Table S2.

**Figure 4:**
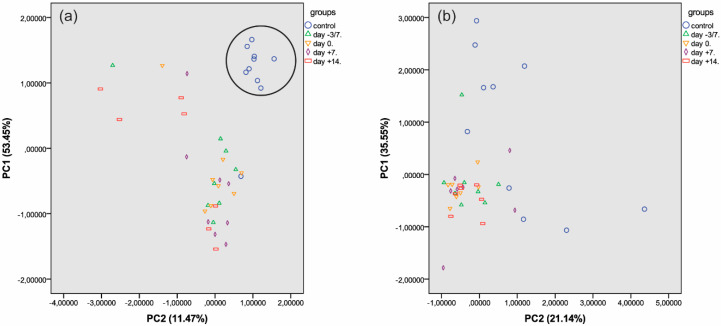
Principal component analysis of serum (**a**) and salivary (**b**) IgA N-glycosylation datasets in controls and at four different stages of APSCT (for key to symbols see upper right corner).

Next, principal component analysis was performed on serum IgA N-glycans. In serum samples, the two principal component axes accounted for 11.47% and 53.45% data variance, respectively, representing 64.92% of data variance cumulatively, which was sufficient to resolve the data into two distinct groups (controls and patients). In the patient group, there was no separation of the four transplantation stages into distinct statistical groups (Figure 4a).

### 3.5. Comparison of Salivary IgA N-glycome Profiles in Healthy Controls and Patients Undergoing APSCT

There were six significantly changed salivary IgA N-glycan structures in the control as well as in the patient group at the four stages of transplantation (Figure 3b). None of the structures changed significantly between any two specified time points of the peritransplantation period. The key statistical characteristics (mean and standard errors of the Peak Area%) of the significantly changed salivary IgA N-glycan structures are listed in Table S5.

In saliva, the two principal component axes accounted for 21.14% and 35.55% data variance, respectively, representing 56.69% of data variance cumulatively, which was insufficient to resolve the data either into control and patient groups or into different groups representing the four transplantation stages (Figure 4b). Comparison of serum- and saliva-specific N-glycans revealed higher numbers of neutral and mannosylated structures in the saliva than in the serum.

### 3.6. Sialoform to Neutral Carbohydrate Ratio (SF/NF) in Serum and Saliva

**Figure 5:**
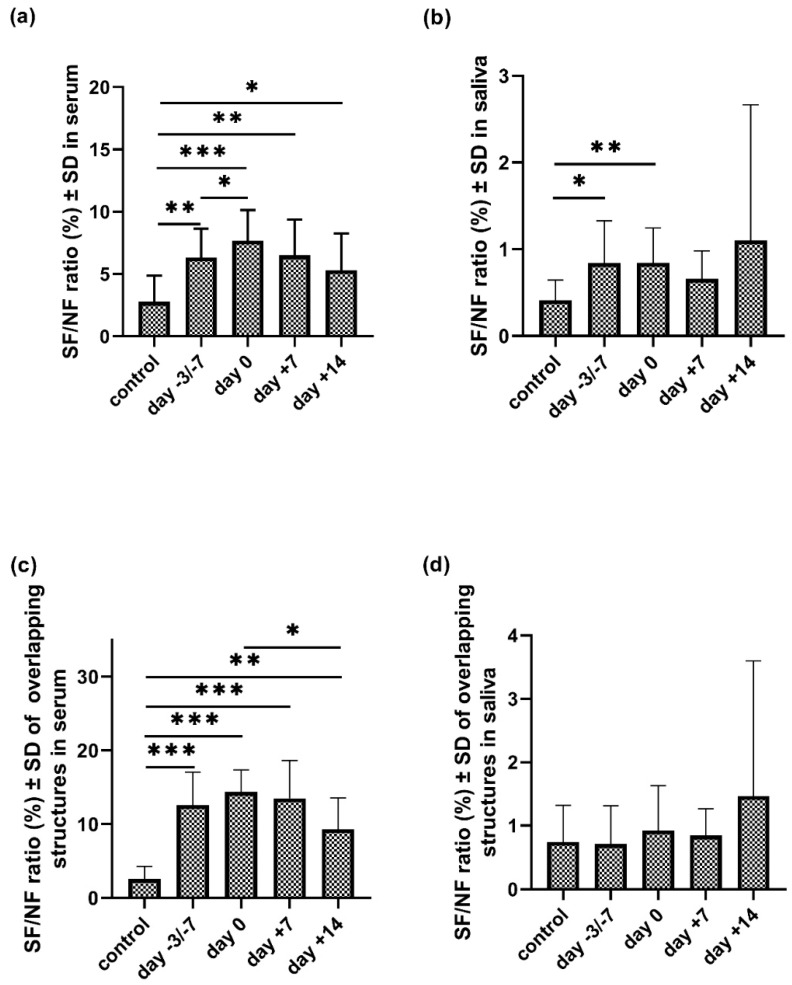
The sialoform to neutral (SF/NF) carbohydrate ratio (%) of serum (**a**) and salivary (**b**) IgA total N-glycans and the overlapping (i.e., present both in serum and saliva) structures (**c**,**d**) in the control and patient group at four stages of autologous peripheral stem cell transplantation (APSCT). Values are expressed as sample means; error bars represent the estimates of standard deviations calculated from three parallel measurements (* *p* ˂ 0.05, ** *p* ˂ 0.01, *** *p* ˂ 0.001).

We calculated the ratios of sialylated and neutral structures in all three possible scenarios (present in serum; in saliva; and in both (i.e., ’overlapping structures’) in the control and patient group at four stages of transplantation (Figure 5, Table S6). This ratio was significantly higher in serum in all examined stages of APSCT as compared to the control group (*p* = 0.002; *p* = 0.001; *p* = 0.002; *p* = 0.043). A significant change of the SF/NF ratio was observed between two specified time points of the transplantation (day −3/−7 and day 0; *p* = 0.05). This ratio was also significantly higher in saliva samples at the day of admission and day 0 compared to the controls (*p* = 0.021; *p* = 0.009). The SF/NF ratio of the overlapping structures in serum was significantly higher in all examined stages of the APSCT compared to the controls (*p* < 0.001; *p* < 0.001; *p* < 0.001; *p* = 0.006) and significantly lower between day 0 and day +14 (*p* = 0.036).

## 4. Discussion

Mucosal barrier injury (oral and enteral mucositis) developing as a result of high-dose intensive cytostatic treatment administered during autologous peripheral stem cell transplantation is often a life-threatening complication. Oral mucositis is a disease of multifactorial ethiopathogenic origin with several patient and treatment related risk factors, which were considered at the study design and exclusion criteria. However, genetic susceptibility was not assessed and we cannot exclude that this could have affected the incidence and severity of mucositis in our patients [15]. Oral mucositis has neither targeted therapy nor biomarkers [9]. Saliva, which contains more than 1000 mostly glycosylated proteins, is a good indicator of changes in plasma constituents’ (hormones, drugs, etc.) and an active subject of biomarker research [16]. Immunoglobulins are glycoproteins with a wide range of functional diversity. Highly glycosylated IgA has the most prevalent role in the mucosal defense [17]. The connection between altered glycosylation of salivary immunoglobulins and oral disease is a rapidly emerging field of research.

### 4.1. Salivary Flow Rate

Several studies have shown that during APSCT, salivary flow rate (both UWS and stimulated whole saliva) decreases because the cytotoxic drugs damage the salivary glands [6,18]. Our results are consistent with these observations. On the day of admission, there was no difference between the control and patient groups, which suggests that the effect of treatments prior to APSCT was not significant compared to the profound effect of high-dose cytostatic drugs. The reserve and regenerative capacity of salivary glands may also play an important role.

### 4.2. Decreased Serum IgA during APSCT

Serum IgA level is affected by several factors, e.g., gender, age, infections. In humoral immuno-deficiencies, including hematological diseases, IgA levels are usually lower than that of the normal population [19,20]. During transplantation, we can observe a further decrease in serum IgA levels, which reach the normal range six months after autologous bone marrow transplantation (BMT) and 12–24 months after allogeneic BMT, unless graft-versus-host disease (GVHD) occurs [8]. In our study, there was a significant decrease in serum IgA during APSCT as compared to both controls and admission day (Figure 2a).

### 4.3. Decreased Salivary IgA Secretion Rate

A significantly reduced level of immunoglobulin subclasses was observed in saliva before APSCT and within five years after [18,21]. Although previous data suggest that sIgA secretion rate decreases in patients with mucositis undergoing chemotherapy compared to patients without mucositis [6], our study revealed no significant correlation between serum IgA and salivary IgA secretion rate and OM degree (Figure 2d,e). Our results indicate that not only the decreased amount but also the altered quality (i.e., glycosylation pattern) of saliva constituents contributes to mucosal barrier injury, increasing the risk and severity of OM.

### 4.4. Analysis of Serum IgA N-glycome Profile

Analysis of the serum IgA N-glycome profile revealed 14 structures with significant differences between the control and patient group at any stage of transplantation. There was one structure, FA2BG2S2, which showed a significant difference between two stages of transplantation (day −3/−7 and day +14) (Figure 3a), a finding worth detailed analysis in the future. In the serum, there was a clear distinction between the control group and the patient group (Figure 4a). This suggests that changes of the serum IgA N-glycan profile can be a disease specific biomarker in APSCT patients. Our results are confirmed by a recent report on altered glycosylation and increased sialylation of IgA1 in the serum of patients with breast cancer, thus stressing its role as a potential biomarker [22].

### 4.5. Analysis of the Salivary IgA N-glycome Profile

The salivary IgA N-glycome profile revealed six structures with significant differences between the control and patient groups at any stage of transplantation. None of these changes have been reported in APSCT yet. Earlier studies reported only on the physiological analysis of the sIgA H chain, J chain and secretory component (SC), and detailed N-glycan and sIgA1 O-glycan profiling [23] and site-specific analysis of salivary IgA [3]. Salivary cytokines and immunoglobulins are important in oral inflammation in malignant hematological disorders, with biomarker potential regarding the efficacy of chemotherapy [24].

Our results suggest that the salivary IgA N-glycome profile reflects the effect of APSCT on local immunity; therefore, it can be suitable for long-term patient follow-up. Serum N-glycome profiles are capable of recognizing differences between control and patient groups. However, this is less pronounced in saliva because it is a sensitively changing open system with great individual variability. It contains approximately 10^8^ viable microorganisms per ml, many of them are capable of producing diverse exoglycosidases (e.g., *Streptococcus oralis* sialidase), modifying the glycosylation patterns of salivary components [16]. Parallel with the progression of periodontitis, hypogalactosylation of serum and gingival crevicular fluid (GCF) IgG has been observed [25]. Although sIgA is more resistant to bacterial proteases than serum IgA [3], the immunoglobulin-degrading effect of certain periodontal pathogens needs to be considered [26]. Thus, the highly variable cariological and periodontal status and person-specific oral flora can further affect glycosylation of the secreted sIgA [3,9,27,28,29].

At least 95% of salivary pool IgA is produced by the plasma cells (PCs) of salivary glands. A minority can be derived from the local PCs of the periodontium as well as serum through the gingival sulcus (crevicular epithelium) via paracellular passive leakage. sIgA- and IgA-producing B-cells have different glycosylation mechanisms and the expression of glycosyltransferases can be altered by several local factors. This explains the differences in regulatory mechanisms in B-cells in different anatomical sites [3].

Several studies have examined the histomorphological effects of chemotherapy on salivary glands both in solid tumors and in hematological malignancies, which is accompanied by a decrease in IgA-producing plasma cells and impaired transcellular immunoglobulin transport [6]. Thus, we can assume that the pre-treatments used and the high-dose conditioning treatment itself during APSCT contribute to salivary gland degeneration to a variable degree, leading to quantitative and qualitative differences in the produced sIgA. Due to the cytotoxic effects, mucosal barrier integrity is impaired, allowing IgA to enter saliva from serum, which can modify the glycosylation profile of the saliva. Hormonal effects can also be assumed. While there is no substantive difference between the two genders in terms of the amount of serum IgA, the salivary sIgA of fertile women was higher than in men, with a fluctuation parallel to serum estradiol (E2) levels suggestive of a relationship between sIgA secretion and E2 [30]. Furthermore, B cells express estrogen receptors and estrogens play regulatory roles in the galactosylation of IgG [31]. The role of sexual dimorphism on the amount of sialic acids in the gingival crevicular fluid has also been confirmed [32]. Appearance of tear and nasal secretion in the saliva may further modify glycosylation patterns [26]. Several other lifestyle, psychological and environmental factors (physical activity, emotions, smoking, infections) can affect the amount and quality of salivary IgA [33], offering a scope for further studies.

### 4.6. Examination of the Sialoform to Neutral Carbohydrate Ratio (SF/NF) in Serum and Saliva

Asialoglycoprotein receptors in the liver eliminate non-sialylated proteins from serum, leading to a higher ratio of sialylated components in the blood than in the saliva [3], in agreement with our findings. Hypersialylation is a glycosylation modification that correlates with tumor genesis, stage, progression and prognosis [34]. Increased free serum sialic acid occurs both in solid tumors including cancers of the oral cavity [35] and in lymphoma and multiple myeloma [36,37,38]. Changes in both free sialic acid levels and N-glycan associated sialylated structures reflect the effectiveness of cytostatic treatment as a useful marker of pathological processes and therapeutic response [38,39,40]. Accordingly, we examined SF/NF ratios. There was a decrease in neutral glycans and an increase in sialylated structures in the patient group compared to controls at any time point of transplantation, except for the overlapping structures in the saliva. Further studies are needed to correlate this with therapeutic response and disease course.

## 5. Conclusions

Oral mucositis is a frequent and life-threatening complication of the intensive conditioning regimen used for hematopoietic stem cell transplantation. Our results indicate that the serum IgA N-glycan profile can serve as a disease-specific biomarker in APSCT patients, whereas changes in salivary IgA reflect its effect on local immunity, rendering it suitable for monitoring OM progression and therapy response. Considering the lack of reliable biomarkers and proven therapeutic targets in OM, our results are clinically relevant, requiring further studies with more patients and longer follow-up periods. Glycosylation site-specific analysis of IgA isotypes and of the secretory component may also provide further insight into the underlying pathological processes. The relevance of IgA glycosylation patterns in viral and other infections of the oral cavity, enteral and respiratory mucosal surfaces is another very timely topic with an urgent need to elaborate novel therapeutic strategies against aggressive pathogens.

## Supplementary Materials

The following are available online at https://www.mdpi.com/2077-0383/9/6/1747/s1, Table S1: The key demographic and clinical data of the participants, Table S2: Mean and standard errors of the PeakArea% of the significantly changed IgA N-glycan structures in the serum, Table S3: Comparison of various IgA N-glycan structures in serum in different groups and time points with Tukey’s multiple comparisons statistical test, Table S4: Comparison of various IgA N-glycan structures in saliva in different groups and time points with Tukey’s multiple comparisons statistical test. Table S5: Mean and standard errors of the PeakArea% of the significantly changed IgA N-glycan structures in the saliva, Table S6: The sialoform to neutral (SF/NF) carbohydrate ratio of saliva and serum total IgA-associated glycans (upper half) and of the overlapping (i.e., present both in serum and saliva) glycan structures (lower half).

## Abbreviations

ANOVA	one-way analysis of variance
APSCT	autologous peripheral stem-cell transplantation
BEAM	BCNU, etoposide, cytosine arabinoside, melphalan
BMT	bone marrow transplantation
CMR	complete morphologic remission
E2	estradiol
EDTA	ethylenediaminetetraacetic acid
ELISA	enzyme linked immunosorbent assays
FA2BG2S2	core fucosylated, sialylated bisecting biantennary glycan
GCF	gingival crevicular fluid
GVHD	graft-versus-host disease
HL	Hodkin lymphoma
Ig	immunoglobulin
IgA	immunoglobulin A
IgG	immunoglobulin G
sIgA	secretory immunoglobulin A
LPS	lipopolysaccharide
MM	multiple myeloma
NHL	non-Hodgkin lymphoma
OM	oral mucositis
PCA	principal component analysis
PCs	plasma cells
PR	partial remission
SC	secretory component
SF/NF	sialoform to neutral carbohydrate ratio
Z(IgA1)	IgA binding affibody protein derived from the Z-domain of Staphylococcal protein A
UWS	unstimulated whole saliva
VGPR	very good partial remission
WHO	World Health Organization

## References

[1] Ohtsubo K., Marth J.D. (2006). Glycosylation in Cellular Mechanisms of Health and Disease. Cell.

[2] Varki A. (2008). Sialic acids in human health and disease. Trends Mol. Med..

[3] Plomp R., de Haan N., Bondt A., Murli J., Dotz V., Wuhrer M. (2018). Comparative Glycomics of Immunoglobulin A and G from Saliva and Plasma Reveals Biomarker Potential. Front. Immunol..

[4] Corthésy B. (2013). Multi-Faceted Functions of Secretory IgA at Mucosal Surfaces. Front. Immunol..

[5] Leong K.W., Ding J.L. (2014). The Unexplored Roles of Human Serum IgA. DNA Cell Biol..

[6] Rafid M., Saka S., Abdullah H.I. (2009). Assessment of salivary flow rate and secretory immunoglobulin A and oral mucosal changes in acute myeloid leukemia before and after the induction phase of chemotherapy. J. Baghdad Coll. Dent..

[7] Rani D.P., Budiardjo S.B., Suharsini M. (2018). Salivary secretory immunoglobulin a level of children with acute lymphoblastic leukemia in maintenance phase. Asian J. Pharm. Clin. Res..

[8] Hammarström V., Pauksen K., Svensson H., Lönnqvist B., Simonsson B., Ringden O., Ljungman P. (2000). Serum immunoglobulin levels in relation to levels of specific antibodies in allogeneic and autologous bone marrow transplant recipients. Transplantation.

[9] Blijlevens N., Schwenkglenks M., Bacon P., D’Addio A., Einsele H., Maertens J., Niederwieser D., Rabitsch W., Roosaar A., Ruutu T. (2008). Prospective oral mucositis audit: Oral mucositis in patients receiving high-dose melphalan or BEAM conditioning chemotherapy—European Blood and Marrow Transplantation Mucositis Advisory Group. J. Clin. Oncol..

[10] Von Elm E., Altman D.G., Egger M., Pocock S.J., Gøtzsche P.C., Vandenbroucke J.P. (2007). The Strengthening the Reporting of Observational Studies in Epidemiology (STROBE) statement: Guidelines for reporting observational studies. Lancet.

[11] Flight L., Julious S.A. (2016). Practical guide to sample size calculations: An introduction. Pharm. Stat..

[12] Bhattarai K.R., Kim H.R., Chae H.J. (2018). Compliance with saliva collection protocol in healthy volunteers: Strategies for managing risk and errors. Int. J. Med. Sci..

[13] Brandtzaeg P. (2013). Secretory immunity with special reference to the oral cavity. J. Oral Microbiol..

[14] Meszaros B., Kovacs Z., Gebri E., Jankovics H., Vonderviszt F., Kiss A., Simon A., Botka S., Hortobagyi T., Guttman A. (2020). N-glycomic analysis of Z(IgA1) partitioned serum and salivary immunoglobulin A by capillary electrophoresis. Curr. Mol. Med..

[15] Basile D., Di Nardo P., Corvaja C., Garattini S.K., Pelizzari G., Lisanti C., Bortot L., Da Ros L., Bartoletti M., Borghi M. (2019). Mucosal injury during anti-cancer treatment: From pathobiology to bedside. Cancers (Basel).

[16] Marsh P.D., Do T., Beighton D., Devine D.A. (2016). Influence of saliva on the oral microbiota. Periodontology.

[17] Schroeder H.W.J., Cavacini L. (2010). Structure and Function of Immunoglobulins (author manuscript). J Allergy Clin. Immunol..

[18] Van Leeuwen S.J., Proctor G.B., Potting C.M., Ten Hoopen S., van Groningen L.F., Bronkhorst E.M., Blijlevens N.M., Huysmans M.C. (2018). Early salivary changes in multiple myeloma patients undergoing autologous HSCT. Oral Dis..

[19] Gonzalez-Quintela A., Alende R., Gude F., Campos J., Rey J., Meijide L.M., Fernandez-Merino C., Vidal C. (2007). Serum levels of immunoglobulins (IgG, IgA, IgM) in a general adult population and their relationship with alcohol consumption, smoking and common metabolic abnormalities. Clin. Exp. Immunol..

[20] Khanna N.N., Das S.N., Khanna S. (1982). Serum immunoglobulins in squamous cell carcinoma of the oral cavity. J. Surg. Oncol..

[21] Norhagen G., Engström P.E., Björkstrand B., Hammarström L., Smith C.I., Ringden O. (1994). Salivary and serum immunoglobulins in recipients of transplanted allogeneic and autologous bone marrow. Bone Marrow Transplant.

[22] Lomax-Browne H.J., Robertson C., Antonopoulos A., Leathem A.J., Haslam S.M., Dell A., Dwek M.V. (2019). Serum IgA1 shows increased levels of α2,6-linked sialic acid in breast cancer. Interface Focus.

[23] Royle L., Roos A., Harvey D.J., Wormald M.R., Van Gijlswijk-Janssen D., Redwan E.R., Wilson I.A., Daha M.R., Dwek R.A., Rudd P.M. (2003). Secretory IgA *N*- and *O*- Glycans Provide a Link between the Innate and Adaptive Immune Systems. J. Biol. Chem..

[24] Pels E.J. (2017). Oral mucositis and saliva IgA, IgG and IgM concentration during anti-tumor treatment in children suffering from acute lymphoblastic leukemia. Adv. Clin. Exp. Med..

[25] Stefanović G., Marković D., Ilić V., Brajović G., Petrović S., Milošević-Jovčić N. (2006). Hypogalactosylation of Salivary and Gingival Fluid Immunoglobulin G in Patients with Advanced Periodontitis. J. Periodontol..

[26] Kerr M.A. (1990). The structure and function of human IgA. Biochem. J..

[27] Marcotte H., Lavoie M.C. (1998). Oral Microbial Ecology and the Role of Salivary Immunoglobulin A. Microbiol. Mol. Biol. Rev..

[28] Laheij A.M., de Soet J.J., Peter A., Kuijper E.J., Kraneveld E.A., van Loveren C., Raber-Durlacher J.E. (2012). Oral bacteria and yeasts in relationship to oral ulcerations in hematopoietic stem cell transplant recipients. Support Care Cancer.

[29] Zaric S.S., Lappin M.J., Fulton C.R., Lundy F.T., Coulter W.A., Irwin C.R. (2017). Sialylation of *Porphyromonas gingivalis* LPS and its effect on bacterial–host interactions. Innate Immun..

[30] Gomez E., Ortiz V., Saint-martin B.L., Boeck L., Díaz-sánchez V.I., Bourges H. (1993). Hormonal regulation of the secretory IgA (sIgA) system: Estradiol- and progesterone-induced changes in sIgA in parotid saliva along the menstrual cycle. Am. J. Reprod. Immunol..

[31] Ercan A., Kohrt W.M., Cui J., Deane K.D., Pezer M., Yu E.W., Hausmann J.S., Campbell H., Kaiser U.B., Rudd P.M. (2017). Estrogens regulate glycosylation of IgG in women and men. JCI Insight.

[32] Nicolau J., Paiva Y.A. (1979). Sialic acid concentration in the attached gingival tissue of rats related to age and sex. J. Periodontal Res..

[33] Deinzer R., Kleineidam C., Stiller-Winkler R., Idel H., Bachg D. (2000). Prolonged reduction of salivary immunoglobulin A (sIgA) after a major academic exam. Int. J. Psychophysiol..

[34] Rodrigues E., Macauley M.S. (2018). Hypersialylation in Cancer: Modulation of Inflammation and Therapeutic Opportunities. Cancers (Basel).

[35] Joshi M., Patil R. (2010). Estimation and comparative study of serum total sialic acid levels as tumor markers in oral cancer and precancer. J. Cancer Res. Ther..

[36] Crook M.A., Couchman S., Tutt P. (1996). Serum sialic acid in patients with multiple myeloma. Br. J. Biomed. Sci..

[37] Nicol B.M., Prasad S.B. (2002). Sialic acid changes in Dalton’s lymphoma-bearing mice after cyclophosphamide and cisplatin treatment. Braz. J. Med. Biol. Res..

[38] Tomaszewska R., Sonta-Jakimczyk D.A., Dyduch A., Olejnik I., Mazur B. (1997). Sialic acid concentration in different stages of malignant lymphoma and leukemia in children. Acta Paediatr. Jpn. Overseas Ed..

[39] Dedova T., Braicu E.I., Sehouli J., Blanchard V. (2019). Sialic Acid Linkage Analysis Refines the Diagnosis of Ovarian Cancer. Front. Oncol..

[40] Kovacs Z., Simon A., Szabo Z., Nagy Z., Varoczy L., Pal I., Csanky E., Guttman A. (2017). Capillary electrophoresis analysis of N-glycosylation changes of serum paraproteins in multiple myeloma. Electrophoresis.

