# Supplementary material for: N-Glycosylation Alteration of Serum and Salivary Immunoglobulin A Is a Possible Biomarker in Oral Mucositis
Source: J Clin Med. 2020 Jun 5;9(6):1747. doi: 10.3390/jcm9061747 (PMC7355945; doi:10.3390/jcm9061747)
Supplement: Supplementary file 1 [file jcm-09-01747-s001.pdf]

Supplementary Material Online

N-glycosylation alteration of serum and salivary immunoglobulin A is a possible biomarker in oral mucositis

Enikő Gebri [1,†], Zsuzsanna Kovács [2,3,†], Brigitta Mészáros [2,3], Ferenc Tóth [4], Ádám Simon [2], Hajnalka Jankovics [3], Ferenc Vonderviszt [3], Attila Kiss [5], András Guttman [2,3,‡] and Tibor Hortobágyi [6,7,8,*,‡]

Table S1. The key demographic and clinical data of the participants.

**Average age (years)**	**Conditioning regimen**	
Controls	BEAM	
41.9±18.35	5	
Patients	Melphalan (high dose, ≥200 mg/m²)	3
49.5±14.19		
**Gender**	**Pretreatment time (months)**	
	Hodgkin lymphoma	
Controls	10.5±0.7	
9F, 1M	Non-Hodgkin lymphoma	
Patients	9±4.6	
5F, 3M	Multiple myeloma	
	10±7	
**Diagnosis**	**Pretransplantational stage of the disease**	
Hodgkin lymphoma	Complete remission	
2	5	
Non-Hodgkin lymphoma	Very good partial remission	
3	1	
Multiple myeloma	Partial remission	
3	2	

BEAM (BCNU, etoposide, cytosine arabinoside, melphalan); F (female); M (male).

Table S2. Mean and standard errors of the PeakArea% of the significantly changed IgA N-glycan structures in the serum.

**Glycan**	**Experimental group**					**p value**
	**control (*n* = 10)**	**day -3/-7 (*n* = 8)**	**day 0 (*n* = 8)**	**day +7 (*n* = 8)**	**day +14 (*n* = 7)**	
**FA2BG2S2**	6.13±3.75[a]	12.1±3.23[b]	11.5±2.45[b]	9.12±2.59[ab]	7.25±2.7[b]	0.0006
FA3G3[3]S3	2.67±2.7[a]	15.1±5.67[b]	15.6±6.65[b]	13.1±4.49[b]	12.9±5.07[b]	<0.0001
A1[3]	1.92±3.41[a]	3.39±1.42[ab]	3.61±1.45[ab]	4.62±1.8[b]	3.4±2.69[ab]	0.0142
A2BG2S1	11.6±5.03[a]	1.46±0.32[b]	1.38±0.239[b]	1.04±0.44[b]	1.19±0.551[b]	0.0010
A2	1.01±0.734[a]	9.86±3.71[b]	11.3±2.64[b]	12.3±1.7[b]	7.25±5.99[ab]	0.0002
A2B	2.08±1.59[a]	9.11±2.94[b]	8.53±2.95[b]	7.12±2.32[b]	10.3±3.73[b]	0.0001
FA2	0.475±0.277[a]	3.8±1.76[b]	2.97±1.59[b]	4.46±1.88[b]	2.75±1.9[b]	<0.0001
M7b	1.98±0.734[a]	0.598±0.153[ab]	0.354±0.178[b]	0.654±0.7[b]	1.17±0.937[ab]	0.0002
A4	5.97±2.79[a]	2.63±0.638[ab]	1.94±0.751[b]	2.37±1.34[ab]	1.47±1.14[b]	<0.0001
FA2[3]G1	3.28±1.72[a]	0.503±0.184[b]	0.525±0.245[b]	0.496±0.151[b]	0.46±0.273[b]	<0.0001

A2G2	3.92±0.922[a]	0.646±0.384[b]	0.748±0.492[b]	0.678±0.28[b]	1.37±0.761[ab]	<0.0001
A2BG2	1.26±0.437[a]	0.774±0.3[ab]	0.574±0.341[b]	0.705±0.386[ab]	0.671±0.36[ab]	0.0239
FA2G2	10.7±4.59[a]	2.06±1.04 [ab]	1.45±0.86[b]	1.78±0.752[b]	1.82±0.83[b]	<0.0001
FA2BG2	1.79±0.522[a]	0.306±0.434[b]	0.404±0.684[b]	0.153±0.074[b]	0.259±0.273[b]	<0.0001

Values are mean PeakAreas%±SD. Superscript 'a' indicates baseline control level; 'b' indicates significant difference ($p<0.05$), 'ab' indicates no significant difference, analyzed with one-way analysis of variance (ANOVA).

Table S3. Comparison of various IgA N-glycan structures in serum in different groups and time points with Tukey's multiple comparisons statistical test. $p<0.05$ is considered significant difference.

Comparison	FA2BG2S2	FA3G3[3]S3	A1[3]	A2BG2S1	A2
control vs. day -3/-7	0.0016	<0.0001	0.6844	<0.0001	0.0464
control vs. day 0	0.0058	<0.0001	0.5642	<0.0001	0.0069
control vs. day +7	0.2518	0.0008	0.01354	<0.0001	0.0002
control vs. day +14	0.9424	0.0017	0.708	<0.0001	0.2887
day -3/-7 vs. day 0	0.9919	0.9997	0.9997	>0.9999	>0.9999
day -3/-7 vs. day +7	0.2894	0.9314	0.8346	0.9974	>0.9999
day -3/-7 vs. day +14	0.0279	0.9047	>0.9999	0.9996	>0.9999
day 0 vs. day +7	0.5354	0.8589	0.9104	0.9989	>0.9999
day 0 vs. day +14	0.0755	0.8238	0.9998	>0.9999	>0.9999
day +7 vs. day +14	0.7583	>0.9999	0.8552	>0.9999	0.594

Comparison	A2B	FA2	M7b	A4	FA2[3]G1
control vs. day -3/-7	<0.0001	0.0006	0.1235	0.4163	0.0099
control vs. day 0	0.0001	0.0137	0.0001	0.0158	0.0111
control vs. day +7	0.0034	<0.0001	0.0125	0.0699	0.0135
control vs. day +14	<0.0001	0.0382	0.8881	0.0024	0.0038
day -3/-7 vs. day 0	0.9928	0.8194	0.7773	>0.9999	>0.9999
day -3/-7 vs. day +7	0.5898	0.9109	>0.9999	>0.9999	>0.9999
day -3/-7 vs. day +14	0.906	0.6907	>0.9999	>0.9999	>0.9999
day 0 vs. day +7	0.8348	0.3215	>0.9999	>0.9999	>0.9999
day 0 vs. day +14	0.7037	0.9988	0.1746	>0.9999	>0.9999
day +7 vs. day +14	0.1725	0.2297	>0.9999	>0.9999	>0.9999

Comparison	A2G2	A2BG2	FA2G2	FA2BG2
control vs. day -3/-7	0.0004	0.4809	0.0513	0.0062
control vs. day 0	0.0007	0.0301	0.0012	0.0012
control vs. day +7	0.0011	0.1386	0.0176	0.0096
control vs. day +14	0.3979	0.1742	0.0233	0.0216
day -3/-7 vs. day 0	>0.9999	>0.9999	>0.9999	>0.9999
day -3/-7 vs. day +7	>0.9999	>0.9999	>0.9999	>0.9999
day -3/-7 vs. day +14	0.6738	>0.9999	>0.9999	>0.9999
day 0 vs. day +7	>0.9999	>0.9999	>0.9999	>0.9999
day 0 vs. day +14	0.9141	>0.9999	>0.9999	>0.9999
day +7 vs. day +14	>0.9999	>0.9999	>0.9999	>0.9999

Table S4. Comparison of various IgA N-glycan structures in saliva in different groups and time points with Tukey's multiple comparisons statistical test. $p<0.05$ is considered significant difference.

Comparison	FA2	M7a	FA2B	A2G2	Unknown
control vs. day -3/-7	0.0377	0.1754	0.1419	0.0836	0.017
control vs. day 0	0.1232	0.0613	0.0523	0.1095	>0.9999
control vs. day +7	0.0165	0.2695	0.0109	0.2522	>0.9999
control vs. day +14	0.0054	0.0428	0.0109	0.0096	>0.9999
day -3/-7 vs. day 0	0.9993	0.9773	>0.9999	>0.9999	>0.9999
day -3/-7 vs. day +7	0.9723	>0.9999	>0.9999	0.985	0.5102
day -3/-7 vs. day +14	0.9476	0.9473	>0.9999	0.9587	>0.9999
day 0 vs. day +7	0.9346	0.974	>0.9999	0.9818	>0.9999
day 0 vs. day +14	0.8952	0.9999	>0.9999	0.9769	>0.9999
day +7 vs. day +14	>0.9999	0.9446	>0.9999	0.7404	>0.9999

Comparison	Unknown
control vs. day -3/-7	0.0939
control vs. day 0	0.0336
control vs. day +7	0.6541
control vs. day +14	0.0747
day -3/-7 vs. day 0	0.9721
day -3/-7 vs. day +7	0.7224
day -3/-7 vs. day +14	0.9987
day 0 vs. day +7	0.3935
day 0 vs. day +14	0.9976
day +7 vs. day +14	0.5993

Table S5. Mean and standard errors of the PeakArea% of the significantly changed IgA N-glycan structures in the saliva.

Glycan	Experimental group					p value
	control (n = 10)	day -3/-7 (n = 8)	day 0 (n = 7)	day +7 (n = 7)	day +14 (n = 7)	
FA2	4.33±2.84^{a}	1.72±0.69^{b}	1.96±1.3^{ab}	1.1±0.334^{b}	1.04±0.918^{b}	0.0030
M7a	6.2±4.41^{a}	3.34±2.01^{ab}	2.57±1.12^{ab}	3.43±1.48^{ab}	2.37±1.21^{b}	0.0334
FA2B	15.5±5.55^{a}	6.31±6.42^{ab}	3.87±2.46^{ab}	3.79±2.59^{b}	3.3±1.97^{b}	<0.0001
A2G2	15.7±10.2^{a}	5.15±4.39^{ab}	4.88±4.52^{ab}	7.49±7.01^{ab}	2.19±1.09^{b}	0.0092
Unknown	4.82±2.06^{a}	1.23±0.577^{b}	3.41±2.58^{ab}	6.92±9.29^{ab}	3.34±1.93^{ab}	0.0408
Unknown	4.65±1.84^{a}	1.72±1.08^{ab}	0.905±0.576^{b}	3.22±3.37^{ab}	1.36±0.673^{ab}	0.0157

Values are mean PeakAreas%±SD. Superscript 'a' indicate baseline control level; 'b' indicates significant difference ($p<0.05$), 'ab' indicates no significant difference, analyzed with one-way analysis of variance (ANOVA).

Table S6. The sialoform to neutral (SF/NF) carbohydrate ratio of saliva and serum total IgA-associated glycans (upper half) and of the overlapping (i.e. present both in serum and saliva) glycan structures (lower half).

**Saliva samples**					
**Group**	**control**	**day -3/-7**	**day 0**	**day +7**	**day +14**
SF/NF ratio	0.38	0.74	0.70	0.57	0.64
mean%/%	27.63/72.37	42.54/57.46	41.12/58.88	36.16/63.84	39.12/60.88
**Serum samples**					
SF/NF ratio	2.24	5.68	7.12	5.56	3.96
mean%/%	69.12/30.88	85.03/14.97	87.68/12.32	84.75/15.25	79.82/20.18
**Overlapping structures**					
**Saliva samples**					
**Group**	**control**	**day -3/-7**	**day 0**	**day +7**	**day +14**
SF/NF ratio	0.61	0.59	0.79	0.72	0.80
mean%/%	38.03/61.97	37.11/62.89	44.01/55.99	41.93/58.07	44.57/55.43
**Serum samples**					
SF/NF ratio	2.15	11.27	13.85	12.27	5.30
mean%/%	68.25/31.75	91.85/8.15	93.26/6.74	92.46/7.54	84.13/15.87
